# Robust Regression Analysis of Copy Number Variation Data based on a Univariate Score

**DOI:** 10.1371/journal.pone.0086272

**Published:** 2014-02-07

**Authors:** Glen A. Satten, Andrew S. Allen, Morna Ikeda, Jennifer G. Mulle, Stephen T. Warren

**Affiliations:** 1 Division of Reproductive Health, Centers for Disease Control and Prevention, Atlanta, Georgia, United States of America; 2 Department of Biostatistics and Bioinformatics and Duke Clinical Research Institute, Duke University, Durham, North Carolina, United States of America; 3 Department of Human Genetics, Emory University, Atlanta, Georgia, United States of America; 4 Department of Epidemiology, Emory University, Atlanta, Georgia, United States of America; Universite de Montreal, Canada

## Abstract

**Motivation:**

The discovery that copy number variants (CNVs) are widespread in the human genome has motivated development of numerous algorithms that attempt to detect CNVs from intensity data. However, all approaches are plagued by high false discovery rates. Further, because CNVs are characterized by two dimensions (length and intensity) it is unclear how to order called CNVs to prioritize experimental validation.

**Results:**

We developed a univariate score that correlates with the likelihood that a CNV is true. This score can be used to order CNV calls in such a way that calls having larger scores are more likely to overlap a true CNV. We developed cnv.beast, a computationally efficient algorithm for calling CNVs that uses robust backward elimination regression to keep CNV calls with scores that exceed a user-defined threshold. Using an independent dataset that was measured using a different platform, we validated our score and showed that our approach performed better than six other currently-available methods.

**Availability:**

cnv.beast is available at http://www.duke.edu/~asallen/Software.html.

## Introduction

In any procedure for calling CNVs, there will be false positive calls made. While it may seem clear that CNV calls that are longer and/or feature a larger change in log intensity ratio (LIR) are more likely to be validated, it is not clear how to combine length and LIR information into a single measure that can be used to rank CNV calls. Optimally, such a measure would correlate with the chance that a CNV would be experimentally validated. All current methods of calling CNVs are in some way based on statistical information, *e.g.* based on a p-value for a hypothesis test or a posterior probability from a Bayesian model, to determine whether a series of adjacent probes should be considered a CNV. It is not clear *a priori* that statistical information is the best predictor of whether a CNV will validate, and assessing this proposition is the first goal of our paper.

To develop a univariate measure that predicts experimental validation, we introduce a family of scores of the form 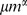, where *μ* is a measure of CNV intensity and *m* is a measure of CNV length. We choose the exponent *α* so that the resulting score is the best predictor of experimental validation. We made this choice using data on log intensity ratios (LIRs) measured using a Nimblegen array comparative genome hybridization (aCGH) platform, with calls made by the Nimblescan software. For a subset of 111 putative CNVs, we used gel electrophoresis of PCR products to determine which calls corresponded to true CNVs.

Because our score is chosen to correlate with the chance a CNV is validated, we wanted to make calls based on this score; in particular, we wanted a fast, easy-to-use algorithm that would call CNVs based on their score, keeping those that exceed a user-specified minimum score. To this end, we developed cnv.beast (backward elimination algorithm with score-based threshold), a novel regression-based computationally-efficient algorithm for calling CNVs.

Although there are numerous algorithms now available for finding CNVs from either array or gene-chip data, few are based on regression. The majority are either change-point algorithms (*e.g.*, circular binary segmentation analysis and its variants) or hidden Markov models (*e.g.*, PennCNV, [1]). We prefer regression to change-point analysis because regression is simple and easily implemented, while change-point analysis is difficult. We prefer regression to hidden variables models because of computational efficiency, and also because hidden variables models require parametric assumptions that are unlikely to be true. In particular, the assumption of independent errors made in a hidden variables model is untrue for high-density data; the effect of assuming independent errors when errors are actually correlated is to underestimate the null probability that a run of adjacent values are elevated, a potentially serious error when trying to call CNVs. Additional methods for calling CNVs include wavelet-based methods [2], smoothing approaches [3], and hierarchical clustering [4]. Additional approaches are described by [5] and [6].

Three regression-based algorithms for finding CNVs are currently available: GLAD [7], a 1-dimensional version of a smoothing-based non-parametric regression approach developed for analyzing 2-dimensional images, and two approaches based on the Lasso [8], [9]. In our experience, the parameters for GLAD are hard to tune and do not have simple interpretations; further, GLAD is computationally intensive. The Lasso-based approach also has several drawbacks. First, the choice of smoothing parameters can be ad-hoc [8] or complex [9], leading to limitations on the number of probes that can be fit [5]. Further, it is not clear that the global optimization criterion used by the lasso corresponds to a good choice of CNVs. For example, small shifts in intensity over a large number of probes may be selected by the lasso but are unlikely to correspond to CNVs. Thus we seek an algorithm tailored to the problem of CNV detection.

The remainder of the paper is organized as follows. We first analyze a set of experimentally-validated calls made using Nimblegen data on the X-chromosome to determine a score function that correlates with the chance that a called CNV overlaps with a true CNV. We then develop cnv.beast, a novel backward-elimination regression algorithm that keeps CNVs having scores that exceed a user-defined threshold. Finally, we validate our approach by using data on deletions in eight Hapmap samples that have been experimentally determined [10]. Ely [5] compared the ability of six previously-published methods to use data from the Illumina 1M chip to detect the CNVs found by Kidd et al. [10]. By analyzing these data with our algorithm, we can assess the performance of our approach relative to existing algorithms for calling CNVs.

### Ordering CNVs by a Score that Predicts Validation

We assume that the observed data comprise the log-intensity ratio (LIR) values at a series of probes having known position in the genome, either from an aCGH experiment or from quantitative intensity data from a genotyping platform (*i.e.*, Illumina or Affymetrix), compared to a reference population. Suppose that from these data, a set of putative CNVs have been proposed. For each called CNV, let *m* denote a measure of the ‘length’ of the CNV (here we use the number of probes that comprise the CNV) and let *μ* denote a measure of the intensity or ‘height’ of a CNV (here we use the absolute value of the median LIR across probes that comprise the CNV). We seek a univariate score of the form 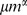 to assign each putative CNV. The choice 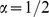 corresponds to statistical information [6], in that a statistical hypothesis test (*e.g.*, a *t*-test) of whether the intensities of the probes comprising the CNV are significantly different from zero would be proportional to 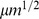. We wish to choose *α* so that high-scoring CNVs have a greater chance of being validated (true).

To choose the value of *α* for the score, we used data on copy number variation on the X chromosome for 41 human males whose DNA is available through the Autism Genetics Resource Exchange (AGRE) [11]. The copy number status of each individual’s X chromosome was queried using three non-overlapping but contiguous Nimblegen comparative genome hybridization (CGH) sub-arrays. Each sub-array had approximately 700,000 probes, so that a LIR was measured at 2,020,823 probes on the X chromosome for each individual. The X chromosome sequence was repeat masked and the PAR1 and PAR2 regions were removed prior to probe selection. This resulted in an average intermarker distance of 50 base pairs or 20 probes/kilobase.

Copy number variants were called using the NimbleScan (NS) software package version 2.4, an implementation of circular binary segmentation analysis, distributed by Nimblegen. Each sub-array was analyzed separately. Data from non-unique probes as well as data from approximately 5% of poorly-behaving probes having unusually large variance was discarded. Spatial correction and normalization were performed using NS, then segment boundaries were determined using the default parameters (no minimum difference in LIR that segments must exhibit before they are identified as separate segments; two or more adjacent probes required to call a change in LIR; maximum stringency for selecting initial segment boundaries).

The NS package gives a list of segment boundaries; because change in LIR across boundaries may be negligible, segment boundaries do not necessarily correspond to CNVs. We selected as CNVs those segments for which the absolute value of the mean LIR was greater than the absolute value of the sum of the mean LIR for the sub-array LIR plus one standard deviation. To identify a parsimonious set of segments for validation, CNVs were merged if their endpoints were within 3kB and if their mean LIRs had the same sign. The LIR of the merged CNV was taken to be the weighted average of the unmerged LIRs, weighted by the number of probes. Finally, to increase reliability, CNVs were only called if the probe density was greater than 9 probes/kB (*i.e.*, slightly less than half of the average probe density for these data, 20 probes/kB).

Using Nimblescan as described above, we obtained 414 putative CNVs. Experimental determination of validation status using PCR amplification followed by gel electrophoresis was successfully completed for 111 putative CNVs called among 41 persons. For generalizability to multiple platforms, we quantile normalized the LIR data before further analysis. Based on examination of both the X-chromosome data described above and data from Affymetrix arrays (data not shown), we chose to quantile normalize to a *t* distribution with 5 degrees of freedom, scaled so that the median absolute deviation (MAD) was 0.2. For each called CNV we counted the number of probes *m* and took the intensity *μ* to be the absolute value of the median LIR for the quantile-normalized data.

We fit a logistic regression model with validation status (*V* = 1 if validated, *V* = 0 if not) as the outcome, using the log of the absolute intensity ln(*μ*) and the log of the number of probes ln(*m*) as predictor variables, *i.e.*

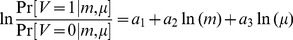
(1)


CNVs found in the same individual as well as overlapping CNVs found in multiple individuals were treated as independent when fitting this model. The region of *μ* and *m* values where *V* = 0 is more likely and the region of *μ* and *m* values where *V* = 1 is more likely is separated by the decision boundary where 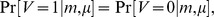 which corresponds to the line
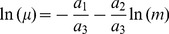
(2)


Note that for any scoring function of the form 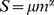, contours of constant score are also straight lines of the form (2) with 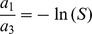 and 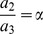. Scoring based on statistical information thus corresponds to 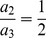.

**Figure 1:**
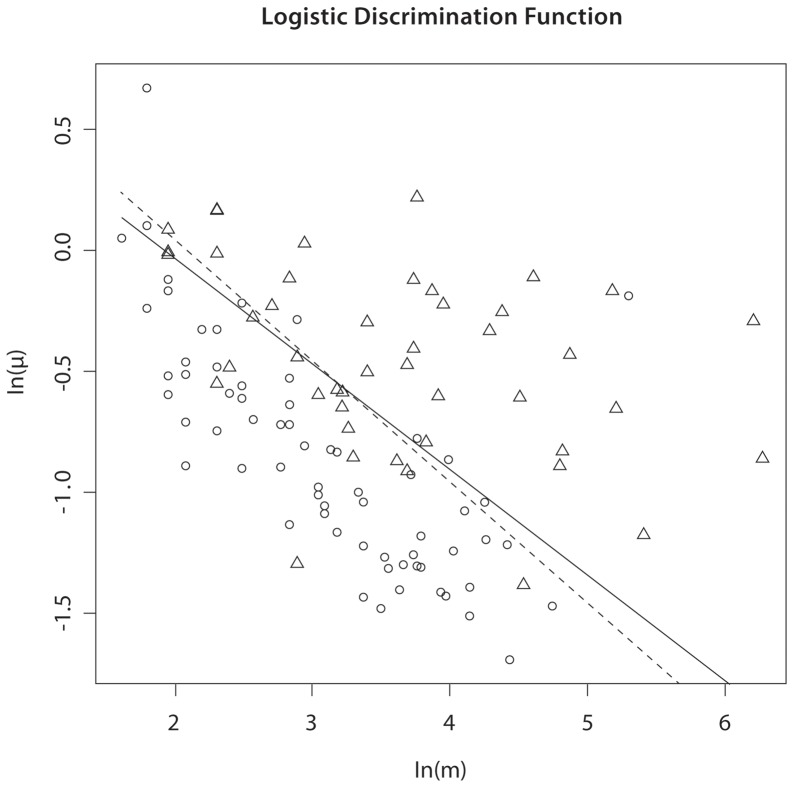
Logistic discrimination functions for Nimblegen X-chromosome data corresponding to optimal score (*α* = 0.44, solid line) and statistical information score (*α* = 0.5, dashed line). Triangles correspond to true positives, ellipses to false positives.

By fitting the logistic model, we found 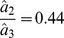 and that a 95% confidence interval for 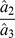 obtained using the delta method (on the log scale) was (0.32, 0.59). Figure 1 shows a plot of validation status by ln(*μ*) and ln(*m*), with the logistic regression discrimination function (2). Visual examination of Figure 1 suggests that a scoring function of the form 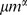 is valid, as the proportion of validated CNVs increases perpendicular to lines of constant score. Note that the choice 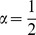 lies in the confidence interval for *α* and is thus consistent with these data. In Figure 1 we also plot the discrimination function obtained by fitting model (1) subject to the restriction 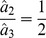. Visual examination of Figure 1 shows that the restricted model predicts experimental validation almost as well as the unrestricted model. Thus, statistical information as measured by 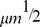 correlates with experimental validation, and for all subsequent analyses in this paper, we used the choice 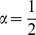.

### A Regression Approach to CNV Calling using a Backward Elimination Algorithm with a Score-based Threshold

We describe cnv.beast, a novel backward elimination algorithm for regression analysis of CNV data that is based on the availability of a univariate score function for ordering CNVs. The algorithm is based on a regression model in which LIR data from an individual is regressed on a series of step functions having jumps at each probe. We treat each step function (jump) as a CNV (with length given by the distance to the nearest jump and height given by the change in predicted magnitude) so that a score for each term can be calculated. Then, the algorithm implements backward elimination until each term in the regression model has a score higher than a user-specified cutoff *S* ^*^, while also eliminating CNVs that contain fewer than *m_min_* probes or have intensity less than µ*_min_*. Any univariate scoring function can be used; here we use 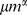 with 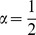.

We use backward elimination to avoid masking. Masking occurs in forward selection algorithms when a term that would correspond to one boundary of a CNV is not entered into the model because the term that corresponds to that CNVs other boundary is not yet in the model. For example, a CNV comprised of probes 80–120 would be described by two terms in equation (3): 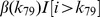 and 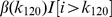. A forward selection algorithm that adds these terms one at a time may find that neither term should be added by itself. By using backward elimination, and by starting with a possible term at each probe, we hope to avoid masking.

We advocate quantile normalization of the log intensity ratios even if the numerator and denominator have already been normalized, so that the same cutoffs can be used for all datasets. As described previously, we normalized to a student t distribution with 5 degrees of freedom, scaled so that the median absolute deviation was 0.2. We made this choice so that our cutoffs for quantile normalized data could also be reasonably applied to untransformed data if necessary.

### Regression Analysis of CNV Data

Let 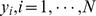 denote the log intensity ratio for data on *N* probes from a single chromosome or chromosomal subregion for a single individual. The goal of our analysis is to fit step functions to the *y_i_*s to determine the locations of the jumps (places where the copy number may change) and the magnitude of these changes. We assume that the (normalized or centered) *y_i_* can be described using the model
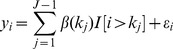
(3)where *k_j_* is the location (probe number) of the *j*th of *J* change points, *β*(*k_j_*) is the change in log intensity ratio between probe *k_j_* and *k_j_*+1 and 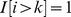 if *i>k* and 0 otherwise. Our goal is to select the change points *k_j_* and the values *β*(*k_j_*). We denote the resulting step function fit to the data *y_i_* by
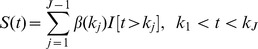



While it is possible to determine fit by using least squares, *i.e.* by minimizing
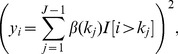
(4)we instead propose a robust regression approach that minimizes
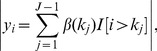
(5)which is robust to isolated large values that are present in CNV data even after quantile normalization.

When 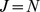 and consequently 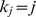, the model is saturated and has 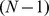 jumps (*i.e.*, takes a different value between each probe). This model is clearly over-fit. When using least squares, one approach to thinning the set of jumps is to use the Lasso, which corresponds to minimizing the saturated model (4) subject to the constraint that 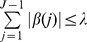 for some appropriately chosen smoothing parameter *λ* [2], [9]. Here we adopt a different approach which is specifically tailored to the CNV problem, is computationally efficient when using (5), and features a novel backward-elimination algorithm that allows control of the intensity, length and score of CNVs that are detected.

Our backward elimination algorithm begins with the saturated model 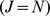 having 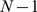 terms, and removes one term from (3) at each step. Thus, at the beginning of the *r*th step, there are 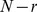 terms in the model; we denote the probes that are in the model at the start of the *r*th step by 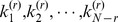. At each step, we remove a single jump, *i.e.* we remove a single value 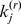 from the set of jumps.

Backward elimination is facilitated by the following observations. First, the values of *β*(*j*) that minimize either (4) or (5) for the saturated model are
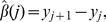
so that the saturated model can be easily fit. Second, at the *r*th step of backward elimination, the least-squares estimator of 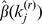 is



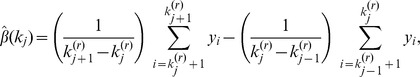
while the L1 estimator of 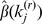 is



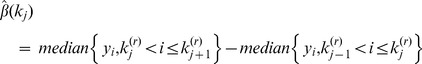
(6)Importantly, note that removing a term from (3), say 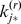, only affects the values of 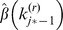 and 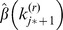. As a result, backward elimination can be carried out very efficiently; for each term removed it is only necessary to update the two adjacent coefficients.

### Backward Elimination using a Score-Based Threshold Algorithm, and the Cutoff Function

We now describe how we choose which jumps to eliminate so that only CNVs having ‘large’ scores are retained. We define the ‘gap’ between the probe 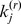 and the nearest probes remaining in the model (i.e., probes for which 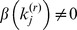) to be 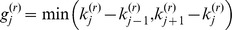 with the convention that 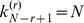 and 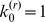. Thus, the *g_j_* is simply the distance to the nearest jump. We wish to eliminate terms for which the change in intensity is ‘small,’ considering the size of the gap. Noting that *β*(*k_j_*) is the magnitude of the change in intensity at probe *k_j_*, we therefore wish to keep the jump at *k_j_* only if the score 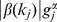 is ‘large.’ This suggests that we keep terms for which
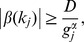
for some value *D*. However, we also wish to ensure that all CNVs that are kept are comprised of at least *m_min_* probes. To avoid jumps of very small magnitude that involve many probes, we also require that CNVs comprised of more than *m_max_* probes also have intensity larger than µ*_min_*. To accomplish all of these goals, we replace the cutoff 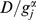 by the cutoff function *C*(*g*), defined by



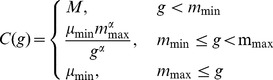
where *M* is some very large number (say, 10^16^) that is much larger than the absolute value of the largest log intensity ratio in the data. The ‘default’ values of the parameters *m_min_*, *m_max_* and µ*_min_* were selected based on our experience with our algorithm, and are given in Table 1. Users may vary these parameters in our software implementation, if they so desire.

**Table 1:** Choice of Parameters in Cutoff Function.

Parameter	Default Value
*m_min_*	6
*m_max_*	30
µ*_min_*	0.25
*α*	0.5

Having chosen the form of the cutoff function *C*(*g*), we take as the goal of our algorithm that, at termination, we should have
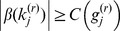
(7)for all terms remaining in the model. To this end, we define



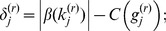



note that 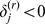 if the jump at 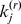 violates (7) and 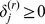 otherwise. Thus, in general, we choose to remove the jump at 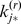 that corresponds to the smallest (*i.e.,* the most negative) value of 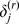. When 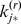 is removed, 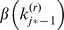, 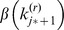, 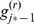, 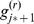, 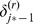, and 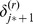 are updated, concluding the *r*th step of the algorithm. The algorithm is terminated at the first step *r* ^*^ for which 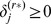 for 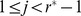, at which point all remaining jumps satisfy (7).

Our backward elimination algorithm can be efficiently executed with a single pass through a sorted list of the values of 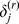. After each step, there are only three values of 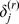 that are out of order; 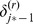, 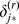 and 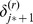 so that it is easy to update the list of sorted values of 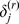 required for subsequent steps of the algorithm.

### Alternative Selection Criterion for Adjacent Jumps in the Same Direction

**Figure 2:**
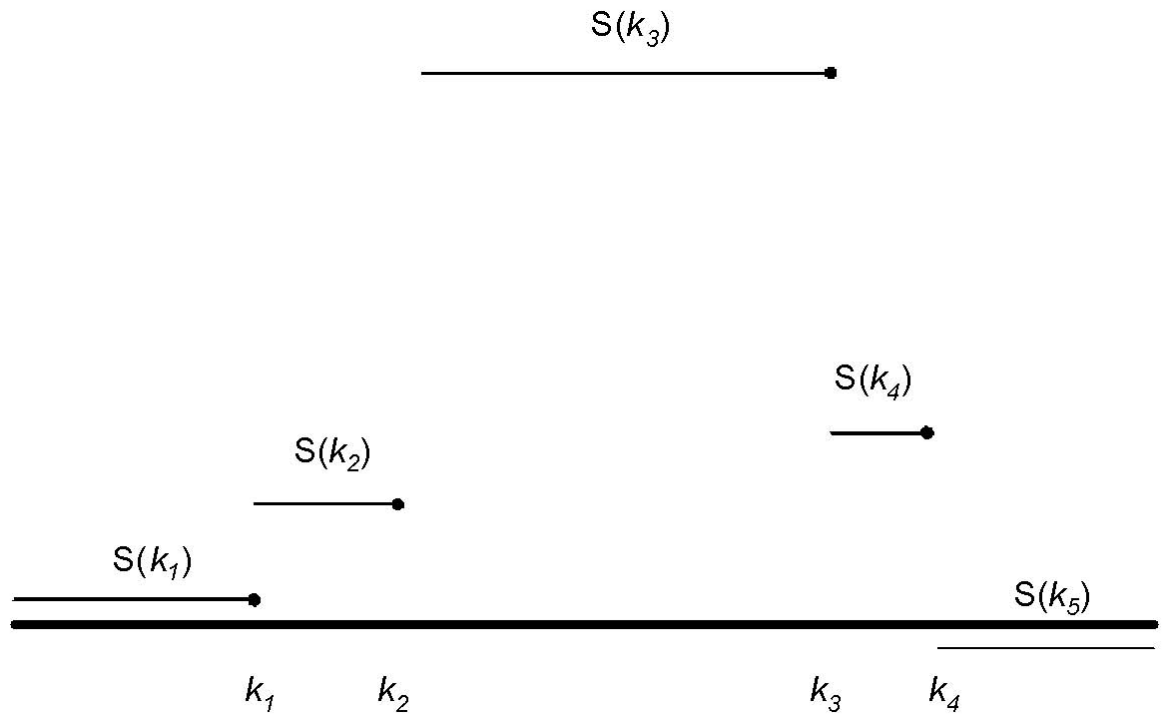
Illustration of situation where removed jump does not have smallest value of *δ*. As illustrated, we would remove the probe at *k*_2_ rather than the probe at *k*_1_, and then the probe at *k*_4_ rather than the probe at *k*_3_ even though 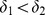 and 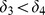 The solid horizontal line corresponds to a log-intensity ratio of 0.

As described above, we choose to remove the jump at 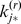 that corresponds to the smallest (most negative) value of 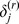. In some situations, as illustrated in Figure 2, this is unwise. Note that for this situation, 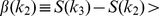 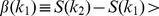 while 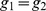 (because the probes at *k_1_* and *k_2_* are each their closest neighbors) so that 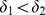, suggesting that we remove *k_2_* before *k_1_*. Similarly, we may be tempted to remove *k_3_* before *k_4_*. However, this may under-estimate the true length of the CNV. Worse, if the (remaining) jumps at *k_2_* and *k_3_* satisfy 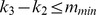, they will be removed and a CNV will not be called. Thus, whenever two adjacent jumps occur in the same direction that take *S*(*k*) further from zero, the first jump will be kept in preference to the second (even if *δ* for the first jump is smaller than the second). Similarly, whenever two adjacent jumps occur in the same direction that result in *S*(*k*) being moved closer to zero, the second jump will be kept in preference to the first (even if *δ* for the second jump is smaller than the first). Formally, these conditions can be stated as follows. When considering whether to remove a probe at position 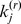, we instead remove the probe at position 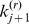 if: (1) 
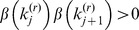
, (2) 
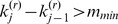
 and 
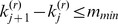
, and (3) 
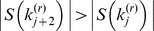
 Similarly, when considering whether to remove a probe at position 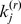, we instead remove the probe at position 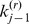 if: (1) 
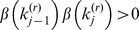
, (2) 
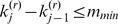
 and 
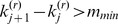
, and (3) 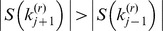.

### Overlapping Blocks for Large Probesets

Although our algorithm is computationally efficient, calculating medians for large numbers of probes between CNVs slows the algorithm as the number of probes *N* increases. To handle datasets with large numbers (∼200,000) of probes, we have developed a variant of our algorithm that breaks the calculation into overlapping blocks of *M* probes. The *m*th such block comprises data *y_i_* on probes 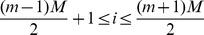 for 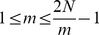; a final block comprising data *y_i_* on probes 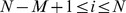 is also used. The algorithm described above is then implemented on each block. Then, the algorithm is restarted using data *y_i_* on all probes, but only allowing terms into model (3) that were retained in at least one of the block analyses.

When *M* is sufficiently large (50,000 probes) we have observed negligible difference in the output of the block and standard versions of our algorithm. We analyzed data on chromosome 2 from 104 individuals, each data set having 148,812 probes, and found no differences in output when using 50,000 (corresponding to 5 blocks) and the analysis done in a single block. The block algorithm can substantially reduce the run time for large *N*. For example, an analysis of 700,000+ probes that took 9½ minutes, when run as a single block, completed in 1½ minutes when run using 15 blocks of 50,000 probes, with identical results. Timings are for a core duo laptop with a 2.53 GHz clock speed and 3 GB RAM.

### The Cleanup Step

**Figure 3:**
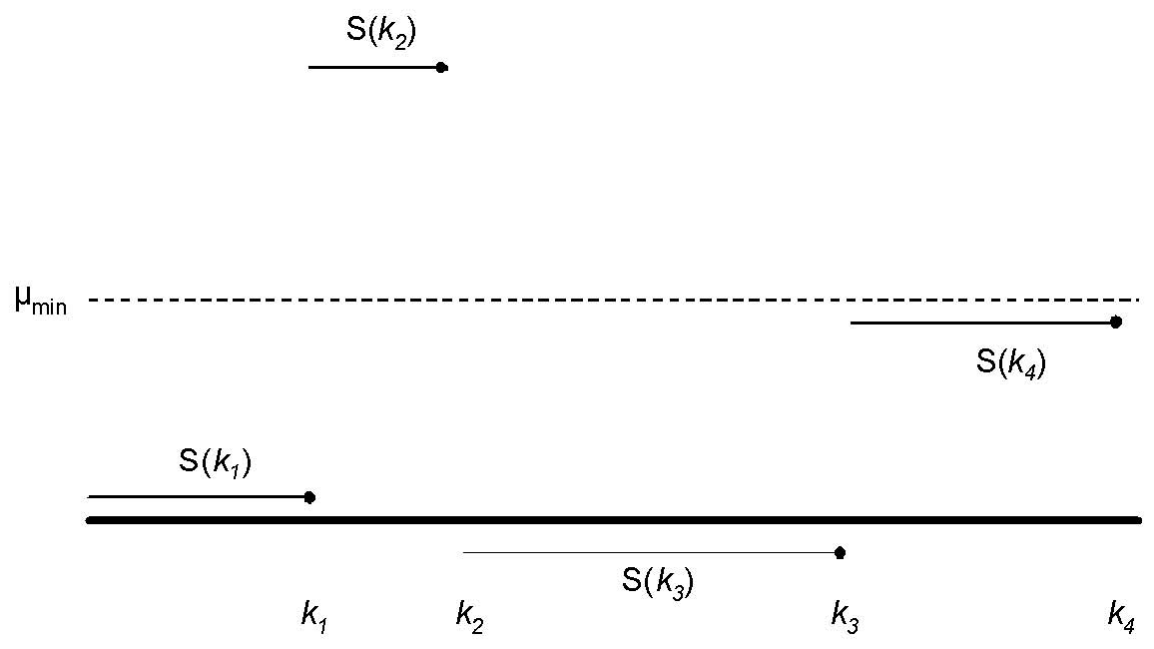
Illustration of cleanup step. As the backward elimination step has terminated, each jump is larger than the appropriate cutoff. At the start of the cleanup step, *S*(*k*_1_) and *S*(*k*_3_) would be set to zero, decreasing 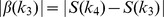 and hence making it a candidate for removal if it becomes smaller than the appropriate cutoff.

At the termination of the algorithm just described (either with or without the use of blocks), the log-intensity ratios predicted by (3) form a step function in which each jump is ‘large enough’ compared with the gap between adjacent retained probes to satisfy our model selection criterion (7). However, because we have not required that the predicted log-intensity ratio return to zero between adjacent CNVs, it can occur that the predicted intensity between probes is actually less than µ*_min_* (see Figure 3). Thus, once the algorithm has terminated, we implement a ‘cleanup step’ in which we re-start the backward elimination (treating the entire data as a single block) with the requirement that all predicted values be either zero or greater than µ*_min_*. This corresponds to replacing (6) with
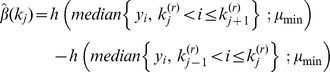
where 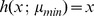 if 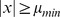 and 0 otherwise.

Finally, even after the cleanup step, some regions may have several jumps before returning to zero. Typically, this occurs for long CNVs. When the predicted LIR has the same sign over the entire region, we use the average intensity over the region, weighted by the number of probes. In those rare cases where a sign change occurs, we consider the probe at which the sign changes to be a boundary between two (adjacent) CNVs. Thus, if a region has first positive and then negative LIR values, we could consider that two CNVs are adjacent; if this occurs, we separately average the predicted intensities over any jumps occurring in regions where the LIR was positive and negative. Scores are then calculated using the length of the region and the averaged intensity.

### Validation using Experimentally Verified Samples

We first applied our algorithm to the Nimblegen data that we used previously to determine the score exponent *α*. Here our goal is to compare the quality of the calls made by Nimblescan to those made by cnv.beast. Of the 111 Nimblescan calls that we have determined validation status experimentally, 44 were found to be true. Using the parameter values in Table 1, cnv.beast detected 88 of the 111 calls; however, of the 23 calls missed by cnv.beast, 17 (74%) failed to experimentally validate (see Table 2). Overall, cnv.beast made 638 calls compared with 414 calls made using our filtering of the calls made by Nimblescan.

**Table 2:** CNV.BEAST calls and Validation Status.

Validation	Detected by CNV.BEAST	
Status:	Yes	No
True	38	6
False	50	17

To assess the performance of our approach in an independent dataset, we analyzed Illumina 1M data from eight Hapmap participants. Deletions among these individuals were determined experimentally by Kidd et al. [10] using fosmid-ESP with additional confirmation by a second method. Deletions in these data were also called by Ely [5] using six CNV-calling programs, allowing us to compare the performance of cnv.beast with previously-existing methods. The methods chosen (and the names of the R packages used) were circular binary segmentation analysis [12] (DNAcopy), hidden Markov partitioning [13] (aCGH), segmentation-clustering [14] (segclust), wavelet segmentation [2] (waveslim), fused lasso segmentation [9] (FLasso), and robust smooth segmentation [3] (smoothseg). The last three methods also utilized the R package ‘cluster’.

**Figure 4:**
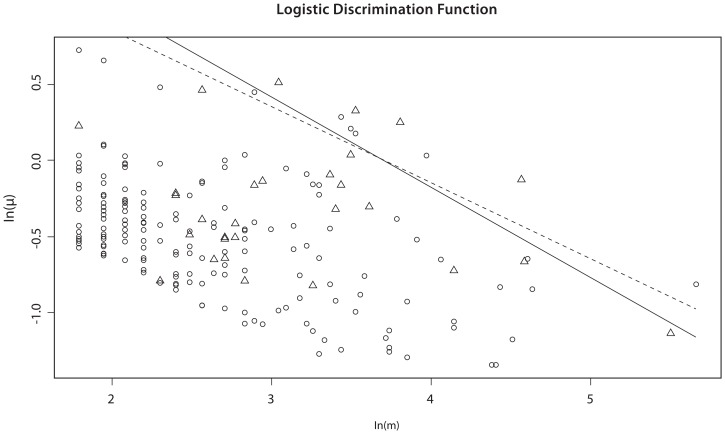
Logistic discrimination functions for Illumina Hapmap data corresponding to optimal score (*α* = 0.59, solid line) and statistical information score (*α* = 0.5, dashed line). Triangles correspond to true positives, ellipses to false positives.

We first validated that our choice of CNV score was predictive of validation in these data by fitting the logistic regression model (1) to quantile-normalized data. We found good agreement between the exponent we obtained using Nimblegen X-chromosome data and the Illumina data; the estimated exponent was 0.59 with 95% confidence interval (0.34, 1.04). In Figure 4 we compare the best-fitting logistic model to the case 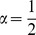. Comparing Figure 4 with Figure 1, we note the higher proportion of false positive calls due to the exhaustive enumeration of deletions in these data compared with the more selective approach taken in the Nimblegen data.

Cnv.beast performed well when compared to the six methods considered by Ely (see Table 3). Details of the implementation of these six methods in these data can be found in Ely (2009). We note first that our algorithm processed data from all eight individuals in 6½ minutes on a core duo laptop with a 2.53 GHz clock speed and 3 GB RAM. Note that cnv.beast had the second smallest number of calls (195) but the highest proportion of true deletions that were at least partially covered by a called region (29.9%). To compare with Ely, we calculated the false discovery rate (FDR) only for calls that exceeded 6000 base pairs in length. Of the 167 calls we made that exceeded this threshold, 138 did not overlap with a true deletion, for a false discovery rate of 82.6%. The only methods with notably lower FDR made fewer true discoveries (9 for SmoothSeg and 17 for SegClust) than the 29 we made.

**Table 3:** Comparison of Sensitivity and FDR.

Method	# of calls	Sensitivity^1^	# of calls >6kbp	FDR^2^
Circular BinarySegmentation	315	0.218	104	0.788
Hidden MarkovModel	20,226	0.287	1,081	0.957
Segmentation/Cluster	837	0.208	55	0.691
Wavelet-basedSegmentation	13,665	0.198	187	0.840
Fused Lasso	655	0.248	130	0.808
RobustSmoothing	37	0.059	29	0.690
CNV>BEAST	195	0.299	167	0.826

1 Sensitivity is the number of true deletions that overlap at least partially with a called deletion, divided by the number of true deletions.

2 FDR is the number of called deletions that do not overlap even partially with a true deletion, divided by the number of called deletions.

**Figure 5:**
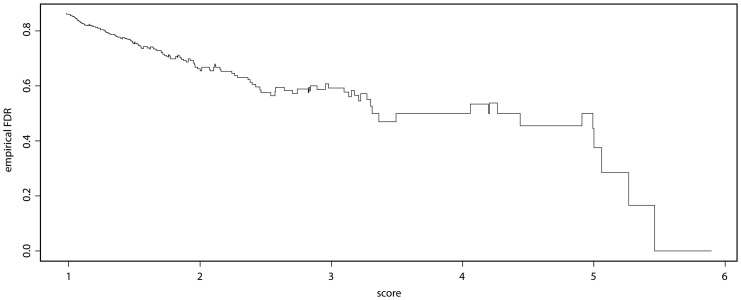
Empirical FDR for calls longer than 6000 base pairs, Hapmap data.

Although the FDR of cnv.beast was 82.6% overall, it is possible to achieve lower FDRs by further filtering the list of called CNVs to those having score greater than some specified value. For example, the FDR for our method among calls made by cnv.beast using the default parameters in Table 1, that further have score greater than 2.5, is 69.2% while the FDR for calls having score greater than 5 is 50%. Considering only calls with score greater than 2.5 where the FDR of our method is comparable to SegClust (17) and SmoothSeg, our method finds 20 true deletions, more than found by either SegClust or SmoothSeg (9). These results suggest that overall, our method outperforms the six competing methods compared by Ely [5]. A plot of the empirical FDR is given in Figure 5. This plot suggests that the score is very useful in prioritizing which calls to experimentally validate.

## Discussion

CNVs are characterized by both height (intensity) and length (number of probes), making it difficult to predict which calls are valid. Using X-chromosome high-density Nimblegen array CHG data, we propose a univariate score that incorporates both intensity data and the number of probes in a call, and that predicts the probability a CNV is valid. We then showed that the same score is a valid predictor of experimental validation in Illumina gene chip data.

Based on the concept of a univariate score, we then developed cnv.beast, a novel backward elimination regression algorithm that keeps terms corresponding to CNVs that exceed a user-defined threshold. Using data from eight Hapmap participants, we showed that cnv.beast had superior performance when compared to the six other methods considered by Ely [5]. Cnv.beast has been successfully used to find CNVs that are risk factors for schizophrenia and autism [15]–[17].

Because our score correlates with the chance of validation, it is a useful quantity to calculate for CNVs called by any method. The plot of FDR as a function of score for the eight Hapmap individuals shown in Figure 5 suggests that the score can be used to prioritize called CNVs for experimental validation. In this context, it is important to quantile normalize data before calculating the score. It is also advantageous to use the median LIR over a region as the measure of intensity, so that calls are not influenced by a single (or a small number of) outlying LIRs.

As a univariate quantity, the score can facilitate Monte-Carlo significance testing. Specifically, if we can generate replicate datasets that are known to have no signal, then for each replicate dataset, we can record the largest score found among all CNVs detected. This distribution can then be used to assign a *p*-value to the CNVs observed in the original data. Hypothesis testing of this type is difficult without a univariate measure to order CNVs. Further, cnv.beast, which is fast even for datasets with many probes, is ideal for this kind of Monte-Carlo analysis. This approach has been implemented by Satten et al. (2012) [18].

Many algorithms for calling CNVs were initially developed for data from cancer cell lines, where copy number changes are often long and hence may be easier to detect. As Table 3 illustrates, the data quality of current CNV platforms is poor when applied to DNA from normal cells, regardless of the algorithm used for calling variants. In order to be useful for association studies (the goal of many studies that use CNVs) it is currently necessary to validate CNV calls using a second technology. Because experimental validation is laborous, slow and expensive, a predictor of validation status such as the score we propose, could be useful in prioritizing which CNV calls to validate, regardless of what algorithm is used to make the calls.

### Software Availability

A fortran program to unleash the power of the beast, as well as an R shell to run it and a pdf file with usage notes, is available at http://www.duke.edu/~asallen/Software.html.

## References

[1] Wang K, Li M, Hadley D, Liu R, Glessner J, et al (2007) An integrated hidden Markov model designed for high-resolution copy number variation detection in whole-genome SNP genotyping data. Genome Research 17: 1665–1674. 17921354 10.1101/gr.6861907 PMC2045149

[2] Hsu L, Self S, Grove D, Randolph T, Wang K, et al (2005) Denoising array-based comparative genomic hybridization data using wavelets. Biostatistics 9: 211–226. 10.1093/biostatistics/kxi004 15772101

[3] Huang J, Gusnanto A, O’Sullivan K, Staaf J, Borg A, et al (2007) Robust smooth segmentation approach for array CGH data analysis. Bioinformatics 23: 2463–2469. 17660206 10.1093/bioinformatics/btm359

[4] Xing B, Greenwood CMT, Bull SB (2007) A hierarchical clustering method for estimating copy number variation. Biostatistics 8: 632–653. 17060368 10.1093/biostatistics/kxl035

[5] Ely B (2009) A comparison of methods for detecting copy number variants from single nucleotide polymporphism intensity data. Masters Thesis, Department of Biostatistics, University of Washington, Seattle WA.

[6] Jeng XJ, Cai TT, Li H (2010) Optimal sparse segment identification with application in copy number variation analysis. Journal of the American Statistical Assocociation 105: 1156–1166. 10.1198/jasa.2010.tm10083 PMC3610602 23543902

[7] Hupe P, Stransky N, Thiery J, Radvanyi F, Barillot E (2004) Analysis of array CGH data. Bioinformatics 20: 3413–3422. 15381628 10.1093/bioinformatics/bth418

[8] Huang T, Wu B, Lizardi P, Zhao H (2005) Detection of DNA copy number alterations using penalized least squares regression. Bioinformatics 21: 3811–3817. 16131523 10.1093/bioinformatics/bti646

[9] Tibshirani R, Wang P (2008) Spatial smoothing and hot spot detection for CGH data using the fused lasso. Biostatistics 9: 18–29. 17513312 10.1093/biostatistics/kxm013

[10] Kidd JM, Cooper GM, Donahue WF, Hayden HS, Sampas N, et al (2008) Mapping and sequencing of structural variation from eight human genomes. Nature 453: 56–64. 18451855 10.1038/nature06862 PMC2424287

[11] Geschwind DH, Sowinski J, Lord C, Iversen P, Shestack J, et al (2001) The Autism Genetic Resource Exchange: a resource for the study of autism and related neuropsychiatric conditions. American Journal of Human Genetetics 69: 463–6. 10.1086/321292 PMC1235320 11452364

[12] Olshen A, Venkatraman E, Lucito R, Wigler M (2004) Circular binary segmentation for the analysis of array-based DNA copy number data. Biostatistics 5: 557–572. 15475419 10.1093/biostatistics/kxh008

[13] Fridlyand J, Snijders A, Pinkel D, Albertson D, Jain A (2004) Hidden Markov models approach to the analysis of array CGH data. Journal of Multivariate Analysis 90: 132–153.

[14] Picard F, Robin S, Lebarbier E, Daudin J (2007) A Segmentation/Clustering Model for the Analysis of Array CGH Data. Biometrics 63: 758–766. 17825008 10.1111/j.1541-0420.2006.00729.x

[15] Mulle JG, Dodd AF, McGrath JA, Wolyniec PS, Mitchell AA, et al (2010) Microdeletions in 3q29 Confer High Risk of Schizophrenia. American Journal of Human Genetics 87: 229–236. 20691406 10.1016/j.ajhg.2010.07.013 PMC2917706

[16] Moreno-De-Luca D, SGENE Consortium, Mulle JG, Simons Simplex Collection Genetics Consortium, Kaminsky EB, et al (2010) Deletion 17q12 is a Recurrent Copy Number Variant that Confers High Risk of Autism and Schizophrenia. American Journal of Human Genetics 87: 618–30. Erratum in: American Journal of Human Genetics 88: 121. 10.1016/j.ajhg.2010.10.004 PMC2978962 21055719

[17] Mulle JG, Pulver AE, McGrath JA, Wolyniec PS Dodd AF, et al.. (2013) Reciprocal Duplication of the Williams-Beuren Syndrome Deletion on Chromosome 7q11.23 is Associated with Schizophrenia. Biological Psychiatry in press. 10.1016/j.biopsych.2013.05.040 PMC3838485 23871472

[18] Satten GA, Ramachandran D, Mulle JG, Allen AS, Bean LJH, et al.. (2012) Testing Copy Number Variant/Trait Associations Detected Using Manhattan Plots. American Society for Human Genetics Abstract # 1349W.

